# Supplementary material for: Identification of photoperiod-induced specific miRNAs in the adrenal glands of Sunite sheep (Ovis aries)
Source: Front Vet Sci. 2022 Jul 22;9:888207. doi: 10.3389/fvets.2022.888207 (PMC9354845; doi:10.3389/fvets.2022.888207)
Supplement: Supplementary file 1 [file Data_Sheet_1.ZIP › Supplementary file/miRDeep2 file.zip!/miRDeep2 file/Novel_Structure/SP42A2/Novel_153.pdf]

Provisional ID	:	NC_019484.2_43583
Score total	:	2.7
Score for star read(s)	:	-1.3
Score for read counts	:	0
Score for mfe	:	2.4
Score for randfold	:	1.6
Score for cons. seed	:	
Total read count	:	312
Mature read count	:	299
Loop read count	:	0
Star read count	:	13

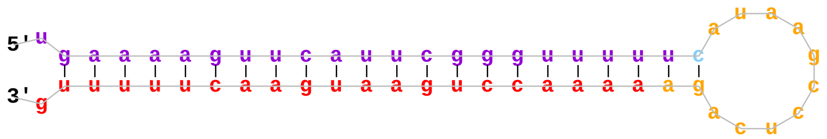

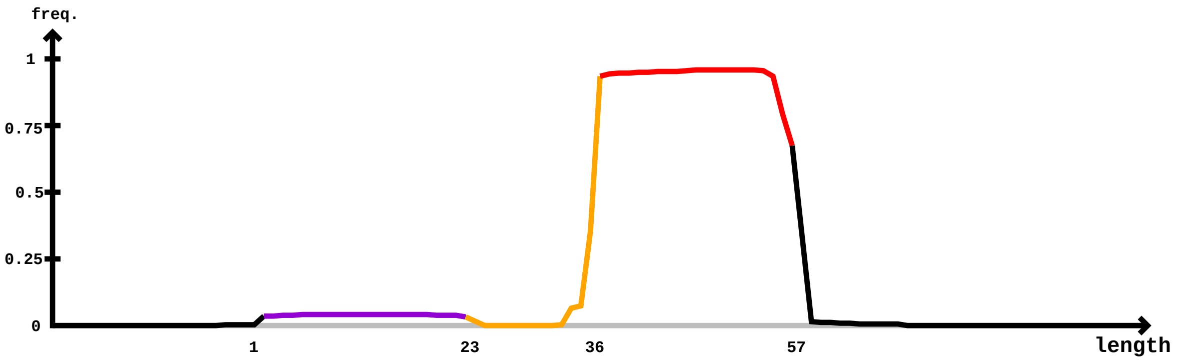

Star Mature

```
5'- cuaucauauauuggguuggcugaaaaguucauucggguuuuucauaagccucagaaaaaccugaaugaacuuuuuggccaacccaauauuaucuaacuaucccaguggaguc   -3'    obs
    cuaucauauauuggguuggcugaaaaguucauucggguuuuucauaagccucagaaaaaccugaaugaacuuuuuggccaacccaauauuaucuaacuaucccaguggaguc          exp
    .......((((((((((((((((((((((((((((((((((((..........))))))))))))))))))))))))))))))))))))..........(((....)))...    reads    mm    sample
    ................uggcugaaaaguucauuUgggu..........................................................................    1    1    seq
    ....................ugaaaaguucauucggguuuu.......................................................................    2    0    seq
    ....................ugaaaaguucauucggguuuuu......................................................................    9    0    seq
    ......................aaaaguucauuUggguuuuuc.....................................................................    1    1    seq
    ........................aaguucauucggguuuuuc.....................................................................    1    0    seq
    ...................................................cagaaaaaccCgaaugaacuuu.......................................    1    1    seq
    ....................................................agaaaaaccugaaugaacuu........................................    1    0    seq
    ....................................................agaaaaaccugaaugaacuuu.......................................    3    0    seq
    ....................................................agaaaaaccugaaugaacuuuC......................................    3    1    seq
    ....................................................agaaaaaccugaaugaGcuuuu......................................    1    1    seq
    ....................................................agaaaaaUcugaaugaacuuuuu.....................................    13    1    seq
    .....................................................gaaaaaccugaaugaacuuuuu.....................................    3    0    seq
    ......................................................Uaaaaccugaaugaacuuu.......................................    1    1    seq
    ......................................................aaaCaccugaaugaacuuuu......................................    1    1    seq
    ......................................................aaaaaccugaaugaacuuuu......................................    6    0    seq
    ......................................................aaaaaccAgaaugaacuuuu......................................    13    1    seq
    ......................................................aaaaaccugaaugaacuuuuC.....................................    1    1    seq
    ......................................................aaaaaccugaaGgaacuuuuu.....................................    6    1    seq
    ......................................................Uaaaaccugaaugaacuuuuu.....................................    1    1    seq
    ......................................................aaaaaccAgaaugaacuuuuu.....................................    2    1    seq
    ......................................................Gaaaaccugaaugaacuuuuug....................................    1    1    seq
    ......................................................aaaaaccugaaugaacuuGuug....................................    2    1    seq
    ......................................................aaaaaccugaaCgaacuuuuug....................................    33    1    seq
    ......................................................aaaaaccAgaaugaacuuuuug....................................    2    1    seq
    ......................................................aaaaaccugaaugaacuuuuug....................................    19    0    seq
    ......................................................Uaaaaccugaaugaacuuuuug....................................    1    1    seq
    ......................................................aaaaaccugaaugaacuuuuugU...................................    1    1    seq
    ......................................................aaaaaccugaaugaacuuuuugg...................................    2    0    seq
    ......................................................aaaaaccugaaugaacuuuuugC...................................    2    1    seq
    ......................................................aaaaaccugaaGgaacuuuuugg...................................    1    1    seq
    .......................................................aaaaccAgaaugaacuuu.......................................    2    1    seq
    .......................................................aaaaUcugaaugaacuuuu......................................    23    1    seq
    .......................................................aaaaccugaaugaacCuuu......................................    1    1    seq
```

Star **Mature**

```
cuaucauauauugguuggcugaaaaguucauucggguuuuucauaagccucagaaaaaccugaaugaacuuuuuggccaacccaauauuaucuaacuaucccaguggaguc
......................................................aaaaccAgaaugaacuuuuu.....................................    9    1    seq
......................................................aaaCccugaaugaacuuuuu.....................................    1    1    seq
......................................................aaaaccugaaugaacuuuuC.....................................    1    1    seq
......................................................Gaaaccugaaugaacuuuuu.....................................    2    1    seq
......................................................aaaaccAgaaugaacuuuuug....................................    2    1    seq
......................................................aaaaUcugaaugaacuuuuug....................................    57   1    seq
......................................................aaaaccugaauAaacuuuuug....................................    1    1    seq
......................................................aaaaccugaaGgaacuuuuug....................................    1    1    seq
......................................................aaaaccugaaugaacuuuuug....................................    18   0    seq
......................................................Gaaaccugaaugaacuuuuugg...................................    3    1    seq
......................................................aaaaccugaaugaacuuCuugg...................................    1    1    seq
......................................................aaaaccugaaugaacCuuuugg...................................    1    1    seq
......................................................aaaaccugaaugaacuuuuugg...................................    56   0    seq
......................................................aaaaccugaaugaacuuuuugA...................................    16   1    seq
......................................................aaaaccugaaugaacuuuuuggG..................................    1    1    seq
.......................................................aaaccugaaugaacuuuuu.....................................    1    0    seq
.......................................................aaaccugaaugaacuuuuugA...................................    2    1    seq
........................................................aaccugaaugaaAuuuuug....................................    1    1    seq
..........................................................ccugaaugaacuuuuuggcca................................    1    0    seq
............................................................uAaaugaacuuuuuggccaac..............................    1    1    seq
...............................................................augaacuuuuuggccaacccaGu.........................    1    1    seq
................................................................ugaacuuuuuggccaacccaaC.........................    1    1    seq
```
